# Whole-genome sequencing of *Listeria innocua* recovered from retail milk and dairy products in Egypt

**DOI:** 10.3389/fmicb.2023.1160244

**Published:** 2023-05-10

**Authors:** Hazem Ramadan, Maha Al-Ashmawy, Ahmed M. Soliman, Mohammed Elbediwi, Islam Sabeq, Mona Yousef, Abdelazeem M. Algammal, Lari M. Hiott, Mark E. Berrang, Jonathan G. Frye, Charlene R. Jackson

**Affiliations:** ^1^Hygiene and Zoonoses Department, Faculty of Veterinary Medicine, Mansoura University, Mansoura, Egypt; ^2^Poultry Microbiological Safety and Processing Research Unit, US National Poultry Research Center, USDA-ARS, Athens, GA, United States; ^3^Department of Food Hygiene and Control, Faculty of Veterinary Medicine, Mansoura University, Mansoura, Egypt; ^4^Department of Microbiology and Immunology, Faculty of Pharmacy, Kafrelsheikh University, Kafr El-Sheikh, Egypt; ^5^Evolutionary Biology, Institute for Biology, Freie Universität Berlin, Berlin, Germany; ^6^Animal Health Research Institute, Agriculture Research Center, Cairo, Egypt; ^7^Department of Food Hygiene and Control, Faculty of Veterinary Medicine, Benha University, Tukh, Qalyubia, Egypt; ^8^Department of Bacteriology, Immunology and Mycology, Faculty of Veterinary Medicine, Suez Canal University, Ismailia, Egypt

**Keywords:** *Listeria innocua*, whole-genome sequencing, genetic context, *clp*L gene, phylogenetic analysis, milk, Egypt

## Abstract

The similarity of the *Listeria innocua* genome with *Listeria monocytogenes* and their presence in the same niche may facilitate gene transfer between them. A better understanding of the mechanisms responsible for bacterial virulence requires an in-depth knowledge of the genetic characteristics of these bacteria. In this context, draft whole genome sequences were completed on five *L. innocua* isolated from milk and dairy products in Egypt. The assembled sequences were screened for antimicrobial resistance and virulence genes, plasmid replicons and multilocus sequence types (MLST); phylogenetic analysis of the sequenced isolates was also performed. The sequencing results revealed the presence of only one antimicrobial resistance gene, *fos*X, in the *L. innocua* isolates. However, the five isolates carried 13 virulence genes involved in adhesion, invasion, surface protein anchoring, peptidoglycan degradation, intracellular survival, and heat stress; all five lacked the *Listeria* Pathogenicity Island 1 (LIPI-1) genes. MLST assigned these five isolates into the same sequence type (ST), ST-1085; however, single nucleotide polymorphism (SNP)-based phylogenetic analysis revealed 422–1,091 SNP differences between our isolates and global lineages of *L. innocua*. The five isolates possessed an ATP-dependent protease (*clp*L) gene, which mediates heat resistance, on a *rep*25 type plasmids. Blast analysis of *clp*L-carrying plasmid contigs showed approximately 99% sequence similarity to the corresponding parts of plasmids of *L. monocytogenes* strains 2015TE24968 and N1-011A previously isolated from Italy and the United States, respectively. Although this plasmid has been linked to *L. monocytogenes* that was responsible for a serious outbreak, this is the first report of *L. innocua* containing *clp*L-carrying plasmids. Various genetic mechanisms of virulence transfer among *Listeria* species and other genera could raise the possibility of the evolution of virulent strains of *L. innocua*. Such strains could challenge processing and preservation protocols and pose health risks from dairy products. Ongoing genomic research is necessary to identify these alarming genetic changes and develop preventive and control measures.

## Introduction

Food animals are considered a major reservoir for human infection with foodborne pathogens such as non-typhoidal *Salmonella*, *Campylobacter* species, *Escherichia coli*, and *Listeria* spp. (Heredia and García, 2018). Genus *Listeria* includes different species that are either pathogenic or non-pathogenic. *Listeria monocytogenes* and *L. ivanovii*, two pathogenic species belonging to *Listeria sensu* strictu, are the primary agents that cause listeriosis in humans and animals, respectively (Schardt et al., 2017). The pathogenicity of these two species is linked to an approximately 9 kb virulence gene cluster (Schmid et al., 2005). Other *Listeria* species, *L. innocua*, *L. seeligeri*, *L. welshimeri*, *L. marthii*, and *L. grayi* are thought to be likely non-pathogenic *Listeria* species (Liu, 2013). Except for *L. grayi*, which belongs to the *Listeria sensu* lato group, all of the presumed non-pathogenic species were classified as *Listeria sensu* strictu and are considered saprophytes (Chiara et al., 2015).

The patterns of genome evolution in *Listeria sensu* strictu and *sensu* lato isolates were investigated in previous sequencing papers (Schmid et al., 2005; den Bakker et al., 2010; Chiara et al., 2015). Surprisingly, these papers documented the acquisition of virulence factors that were lacking in non-pathogenic species of *Listeria sensu* strictu and *sensu* lato at the time they were identified. Gene duplication, gene divergence, and lateral gene transfer—most often from sources outside of *Listeria*—are all common throughout the genus and are presumed to be the underlying mechanisms of bacterial evolution (Chiara et al., 2015).

Despite the fact that *L. innocua* is typically nonhemolytic, a previous study documented the existence of an atypical hemolytic *L. innocua* isolate, which contained every member of the *L. monocytogenes prf*A-regulated virulence gene cluster (*Listeria* pathogenicity island 1) but was avirulent in the mouse pathogenicity test (Johnson et al., 2004). Nevertheless, cases of animal listeriosis caused by *L. innocua* which shared a close genetic relationship with *L. monocytogenes* have been reported (Matto et al., 2022). Therefore, ongoing characterization of atypically pathogenic *Listeria* species, as well as changes in their taxonomy, are critical for development and testing of preservation and processing conditions that prevent their growth and spread in the food industry (Orsi and Wiedmann, 2016).

Whole-genome sequencing (WGS) is now recognized as the most effective approach for genetic characterization of pathogens compared to time-consuming and labor-intensive conventional molecular diagnostic techniques that include isolation, identification, and molecular confirmation of the microbe (Lüth et al., 2018; Uelze et al., 2020). WGS can be used to uncover traits such as those responsible for pathogenicity/virulence, antibiotic resistance, and dissemination of mobile genetic elements. WGS can further be applied to provide a more precise description of the taxonomic differences and phylogenetic relatedness between pathogens through multilocus sequence typing (MLST), clonal complex (CC) determination, core genome MLST (cgMLST) and single nucleotide polymorphism (SNP)-based phylogeny (Ramadan et al., 2021; Stessl et al., 2021; Parra-Flores et al., 2022).

Non-pathogenic *Listeria* spp. particularly *L. innocua*, have been reported in different studies from Egypt from different sources (El-Shenawy et al., 2011; Ismaiel et al., 2014; Dahshan et al., 2016), yet there is a lack of information about its genomic characterization. We aimed in the present study to characterize five *L. innocua* isolates from milk and dairy products. Our goal was to test the emergence of virulence factors and genetic components that enable bacteria to survive, spread, and cause listeriosis.

## Materials and methods

### *Listeria innocua* isolates for whole-genome sequencing

Five *L. innocua* isolates (four from raw milk and one from yoghurt) were chosen from our previous study (Youssef et al., 2020) and subjected to WGS for further genomic characterization. Isolates were recovered from raw milk and dairy products (yoghurt and ice cream) purchased from local supermarkets and retail stores in Mansoura City, Egypt during the period between 2014 and 2018. A 25 mL sample from raw milk or 25 g from dairy products were homogenized with 225 mL of trypticase soy broth (TSB), and the sample-TSB mixture was then processed for culturing on *Listeria* selective media as previously described (Youssef et al., 2020). *Listeria* spp. isolates were submitted to the Bacterial Epidemiology and Antimicrobial Resistance (BEAR) Research Unit, United States Department of Agriculture (USDA), Athens, Georgia, United States, through a Material Transfer Research Agreement between Mansoura University and USDA (Agreement No. 58-6040-0-001-F). Isolates were revived by culture in brain heart infusion (BHI) broth (Becton Dickinson, Sparks, MD, United States) which was incubated for 24 h at 35°C. From incubated BHI broth, 10 μL were streaked onto Modified Oxford (MOX) agar with selective supplement (Oxoid, Basingstoke, Hampshire, United Kingdom) and plates were then incubated at 37°C for 24 h. Characteristic small black *Listeria* spp. colonies were picked from MOX agar and plated onto nutrient agar plates for further biochemical identification using Vitek (BioMérieux, Durham, NC, United States).

### Genomic DNA extraction, DNA library preparation and whole-genome sequencing

Genomic DNA (gDNA) was extracted from *Listeria* isolates using the blood and tissue genomic DNA extraction kit (Qiagen, Germantown, MD, United States); purity of extracted gDNA was assessed using a NanoDrop™ spectrophotometer. The concentration of gDNA was determined using a Qubit^®^ double-stranded DNA (dsDNA) high-sensitivity (HS) assay kit (Life Technologies Inc., Carlsbad, CA, United States) on an Invitrogen Qubit 2.0 Fluorometer (Thermo Fisher Scientific, Waltham, MA, United States) according to manufacturer’s instructions. Sequencing libraries were prepared using Nextera™ XT DNA Sample Preparation Kit and Nextera™ XT Index Kit (Illumina Inc., San Diego, CA, United States). Libraries were sequenced on an Illumina MiSeq platform using a MiSeq v2 reagent kit (Illumina Inc., San Diego, CA, United States) with 500 cycles to generate a paired-end read length of 2 × 250 bp. Quality check of raw reads was determined using FastQC tool,^1^ and low quality reads were trimmed using trimmomatic with the following parameters: leading: 10, trailing: 10, sliding window: 4:20, and minlen: 40. Obtained reads were then *de novo* assembled using A5-miseq assembler (Coil et al., 2015) and assembly statistics that included genome length, number of contigs, coverage, GC% and N50 were determined.

### Bioinformatics analysis

Assembled fasta files were uploaded into Galaxy server^2^ and screened against different databases: ResFinder (Zankari et al., 2012), virulence factor database (VFDB) (Chen et al., 2016), and PlasmidFinder (Carattoli et al., 2014) using the ABRicate (version 1.0.1) tool^3^ for identification of resistance and virulence genes and plasmid replicons, respectively. Further *in silico* analysis was performed using Plasmid SPAdes and PLACNETw tools to separate plasmid contigs from WGS of the examined isolates (Vielva et al., 2017). The reconstructed plasmid sequences were then blasted against the National Center for Biotechnology Information (NCBI) database to determine the closely matched plasmids. A genetic comparison was performed between the reconstructed *L. innocua* plasmids from this study and plasmid sequences retrieved from NCBI using the BLAST Ring Image Generator (BRIG) tool.^4^ Linear comparison to determine the genetic environment of resistance and/or virulence genes located on *L. innocua* plasmids was also determined using the Easyfig^5^ tool (Sullivan et al., 2011). Other mobile genetic elements such as insertion sequences (IS) were searched for using the ISFinder^6^ tool.

To determine MLST types for the examined *Listeria* isolates, the assembled sequences were blasted against the *Listeria* sequence typing database available on BIGSdb.^7^ To put our isolates into context with global lineages of *L. innocua*, a single nucleotide polymorphism (SNP)-based phylogenetic analysis was performed using Snippy v4.4.4^8^ with the following variant calling parameters: minimum base quality 60, minimum read coverage 10 and minimum proportion for variant evidence 0.9. Our five isolates were compared to publicly available genomes (*n* = 260) of *L. innocua* recovered from different sources in the NCBI database (updated April 10th, 2021), and all the enrolled isolates were mapped to the reference *L. innocua* ATCC 33091 genome. Metadata for *L. innocua* sequences retrieved from NCBI database are listed in Supplementary Tables S1, S2. Output files from Snippy that determined SNPs variant calling were combined using Snippy core into a core SNPs alignment. Using the Randomized Accelerated Maximum Likelihood (RAxML) tool, maximum likelihood phylogenetic trees were generated from SNPs alignment, and the trees were then visualized with iTOL (Letunic and Bork, 2016).

## Results

In this study, the 5 *L. innocua* isolates from milk and dairy products were subjected to WGS. Assembly statistics of the sequences of *L. innocua* are listed in Table 1. The genome size was 2.96 Mbp with GC% ranging from 37.2 to 37.3, which is consistent with average genome size and GC% of the complete genome of *L. innocua*. Draft genome sequences of the examined isolates were assembled into an average of 16 contigs (14 contigs for LI-36, 15 for L-I33 and LI-35, 16 for LI-34 and 18 for LI-32), with N50 of 544,812 bp for isolate LI-32, 544,816 bp for isolates LI-34 and LI-35 and 545,607 bp for isolates LI-33 and LI-36.

**Table 1:** Assembly statistics, multilocus sequence typing (MLST), resistance and virulence genes and plasmid replicons of *clpL-*producing *Listeria innocua* recovered from milk and dairy products from Egypt.

Isolates genomic features	LI-32	LI-33	LI-34	LI-35	LI-36
Source/Sample ID	Raw milk/M1	Raw milk/M2	Yoghurt/Y2	Raw milk/M7	Raw milk/M7
Season/year of isolation	Summer/2014	Summer/2014	Summer/2018	Fall/2014	Fall/2014
Genome length (Mbp)	2.96	2.96	2.96	2.96	2.96
No. of contigs	18	15	16	15	14
N50 (bp)	544,812	545,607	544,816	544,816	545,607
Median coverage	115	124	87	90	109
GC content	37.3%	37.2%	37.3%	37.3%	37.2%
MLST	ST-1085	ST-1085	ST-1085	ST-1085	ST-1085
Resistance genes	*fos*X	*fos*X	*fos*X	*fos*X	*fos*X
Virulence genes	*fbp*A, *lap*, *iap*/*cwh*A, *gtc*A, *lpe*A, *lsp*A, *oatA*, *pdg*A, *lpl*A1, *prs*A2, *clp*C, *clp*E, *clp*P	*fbp*A, *lap*, *iap*/*cwh*A, *gtc*A, *lpe*A, *lsp*A, *oatA*, *pdg*A, *lpl*A1, *prs*A2, *clp*C, *clp*E, *clp*P	*fbp*A, *lap*, *iap*/*cwh*A, *gtc*A, *lpe*A, *lsp*A, *oatA*, *pdg*A, *lpl*A1, *prs*A2, *clp*C, *clp*E, *clp*P	*fbp*A, *lap*, *iap*/*cwh*A, *gtc*A, *lpe*A, *lsp*A, *oatA*, *pdg*A, *lpl*A1, *prs*A2, *clp*C, *clp*E, *clp*P	*fbp*A, *lap*, *iap*/*cwh*A, *gtc*A, *lpe*A, *lsp*A, *oatA*, *pdg*A, *lpl*A1, *prs*A2, *clp*C, *clp*E, *clp*P
Plasmid replicon type	*rep*25	*rep*25	*rep*25	*rep*25	*rep*25
Accession No.	JAQHRC000000000	JAQHRB000000000	JAQHRA000000000	JAQHQZ000000000	JAQHQY000000000

fbpA, fibronectin-binding protein; lap, listeria adhesion protein; iap/cwhA, cell wall hydrolase; gtcA, cell wall teichoic acid glycosylation protein; lpeA, lipoprotein promoting entry protein; lspA, lipoproteins-specific signal peptidase II; oatA, O-acetyltransferase gene; pdgA, peptidoglycan N-deacetylase; lplA1, lipoate protein ligase A1; prsA2, post-translocation chaperone; clpCEP, ATP-dependent proteases.

ResFinder analysis of the examined 5 *L. innocua* revealed absence of antimicrobial resistance genes; only *fos*X responsible for intrinsic resistance of *Listeria* spp. to fosfomycin was identified. Screening *L. innocua* contigs using the ABRicate tool in Galaxy against the virulence factor database (VFDB), with a minimum 80% identity and coverage showed the presence of multiple virulence genes belonging to adherence (*fbp*A, and *lap*), invasion (*iap*/*cwh*A, *gtc*A, and *lpe*A), surface protein anchoring (*lsp*A), peptidoglycan modification (*oatA*, and *pdgA*), intracellular survival (*lpl*A1, and *prs*A2) and heat shock proteins (*clp*C, *clp*E, and *clp*P). PlasmidFinder results exhibited the presence of a single plasmid replicon type (rep25) in the examined isolates (Table 1).

**Figure 1:**
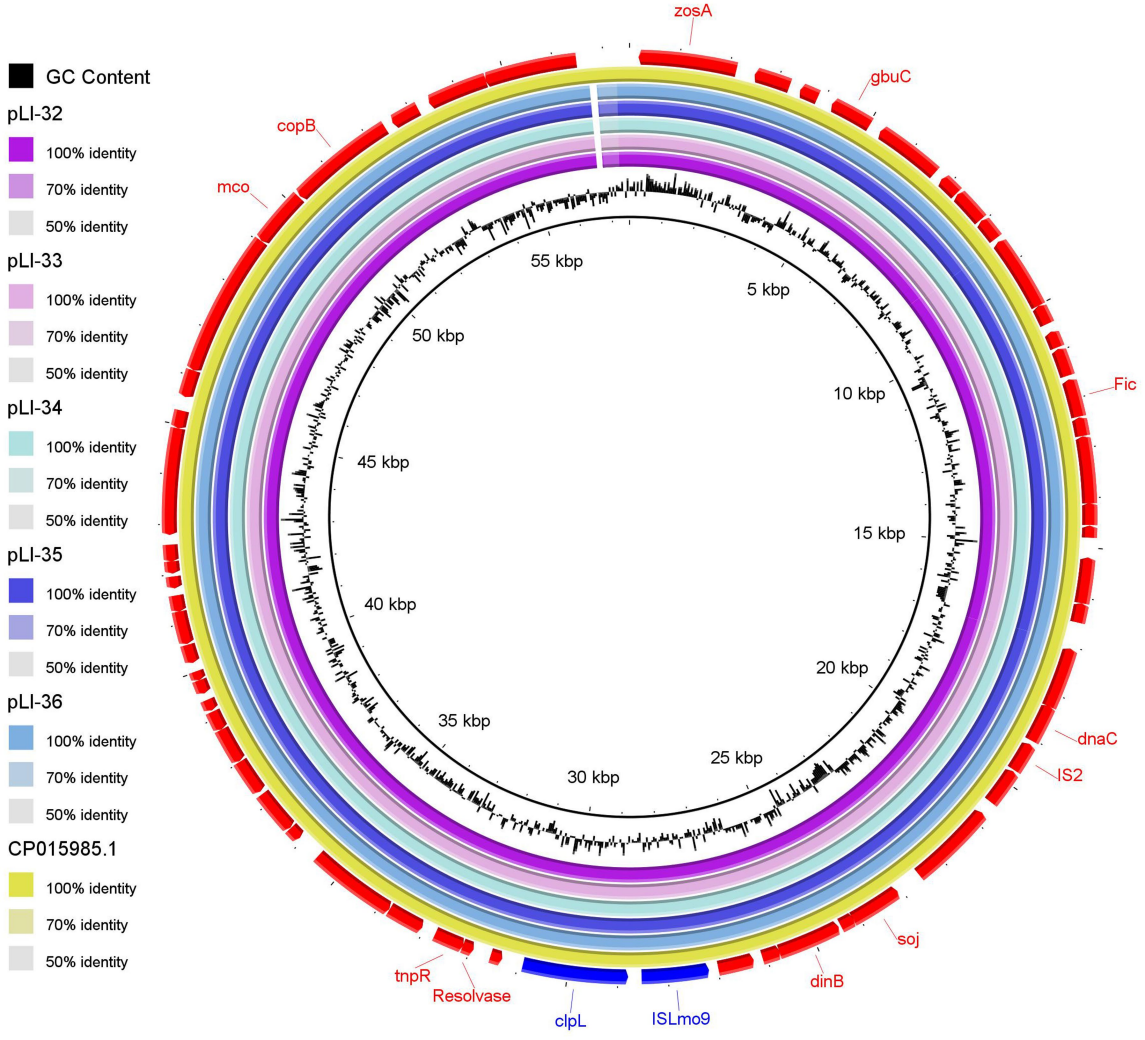
Comparative sequence analysis of the reconstructed plasmids carrying the *clp*L gene from the whole genome sequences of the examined *Listeria innocua* isolates. The reconstructed plasmids pLI-32, pLI-33, pLI-34, pLI-35, and pLI-36 from isolates, were represented as circles from inside to outside. The out-layer circle (red color) represents the reference plasmid (accession no. CP015985.1) used for sequence comparisons. The figure was generated using BLAST Ring Image Generator (BRIG) tool (http://sourceforge.net/projects/brig).

**Figure 2:**
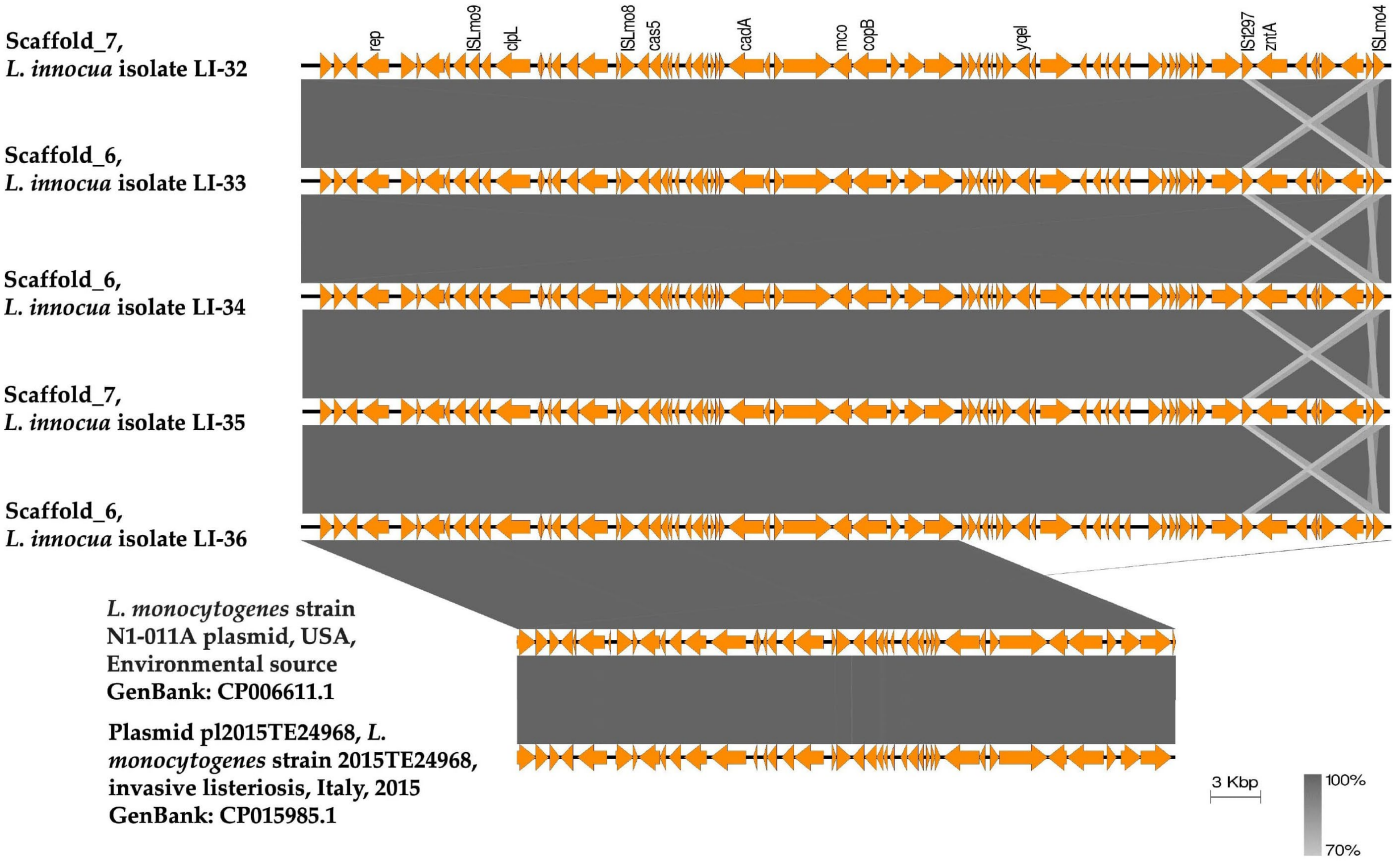
Linear comparison of the plasmid contigs carrying the heat shock *clpL* gene identified from the draft genome sequences of *L. innocua* strains analyzed in this study with a similar plasmid detected from *L. monocytogenes* strain N1-011 in the United States from an environmental source (GenBank: CP006611.1) and plasmid pl2015TE24968 detected from *L. monocytogenes* strain 2015TE24968 isolated from an outbreak of invasive listeriosis in Italy in 2015 (GenBank: CP015985.1). A BLASTN search showed that the plasmid contig carrying the *clpL* gene of the examined *L. innocua* isolates have 99.97% identity with 74% query coverage to the plasmid detected from *L. monocytogenes* strain N1-011, and 99.91% identity with 74% query coverage to plasmid pl2015TE24968. The figure was drawn using the EasyFig tool (http://mjsull.github.io/Easyfig/).

Using plasmid SPAdes and PLACNETw tools, plasmid contigs were distinguished from the draft genome sequences of the examined isolates. Blast analysis of the reconstructed plasmids from our *L. innocua* isolates showed high sequence similarity with plasmids of *L. monocytogenes*: clinical strain 2015TE24968 (accession no. CP015985.1) isolated from an outbreak of invasive listeriosis in Italy in 2015 and environmental strain N1-011A (accession no. CP006611.1) isolated from the United States. Noticeably, the reconstructed *L. innocua* plasmids (pLI-32, pLI-33, pLI-34, pLI-35, pLI-36) carried the *clpL* gene which mediates heat resistance (Figure 1). Blast analysis of *clp*L carrying contigs from our isolates showed approximately 99% sequence similarity to the corresponding parts of plasmids of *L. monocytogenes* strain 2015TE24968 and strain N1-011A. Genetic context of *clpL* in our *L. innocua* and the retrieved NCBI sequences of *L. monocytogenes* revealed the presence of insertion sequence ISLmo8 and ISLmo9 downstream and upstream to the *clpL* gene, respectively (Figure 2).

**Figure 3:**
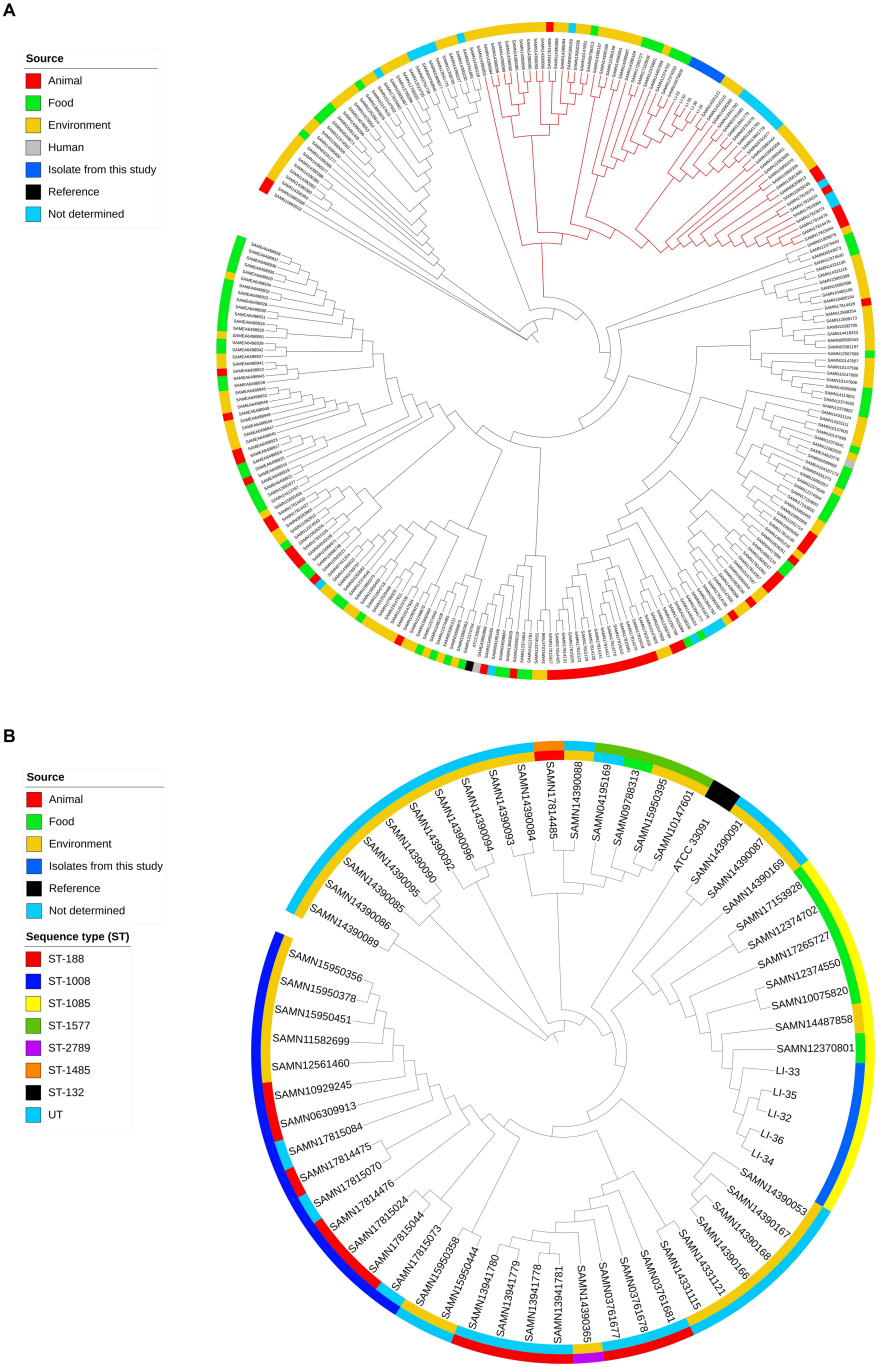
**(A)** Phylogenomic analysis of *Listeria innocua* isolates and publicly available genomes (*n* = 260) of *L. innocua* recovered from different sources in the NCBI database (updated April 10th, 2021); all the enrolled isolated were mapped to the reference *L. innocua* ATCC 33091 genome. The clade with branches in red denotes clustering of closely related NCBI *L. innocua* (*n* = 56) to our isolates. **(B)** Isolates in this clade were further subjected to SNP-based phylogeny for understanding closer genetic relationships among these isolates. From inside-out, the first circle indicates the source of isolates. The second circle indicates the sequence types (STs).

The allelic profile for the seven housekeeping genes retrieved from MLST 2.0 was determined as follows: *abcZ,* 188; *bglA,* 157; *cat,* 182; *dapE,* 223; *dat,* 136; *ldh*, 353; *lhkA*, 148 that assigned the 5 *L. innocua* isolates, regardless of isolate source, into the same sequence type ST-1085. We performed a SNP-based phylogenetic analysis to compare our isolates with the global lineages of *L. innocua*. Findings of SNP-based phylogeny revealed that no specific trends have been observed for clustering isolates based on their source. This was observed from the clustering of examined isolates in a clade with *L. innocua* isolates from different sources, food, and environment (Figure 3A). To zoom in on the differences among isolates within this clade, isolates were separately subjected to a SNP-based phylogeny (Figure 3B). No more than 10 SNP differences were identified among this study isolates, indicating that the five isolates from milk and yoghurt are clonal. The number of SNPs between our isolates and closely related *L. innocua* isolates (SAMN12370801, SAMN12374550, SAMN14487858, SAMN10075820, SAMN17265727, SAMN17153928, and SAMN12374702) ranged from 422 to 1,091 (Supplementary Table S3); where all isolates were assigned to the same ST type (ST-1085) and were sourced from food except isolate SAMN14487858 that was recovered from the environment.

## Discussion

*Listeria* infection is a significant foodborne disease that can be transmitted to humans primarily *via* the food chain. Many food sources are implicated in *Listeria* foodborne infections, including milk and milk products, an important source of protein available to many groups of consumers (Bintsis, 2017; Kim et al., 2018). The pathogenic potential of *L. monocytogenes* is explained by its possession of certain virulence genes that increases bacterial fitness and survivability (Abdelhamed et al., 2022; Osek et al., 2022); however, the pathogenic potentiality of some non-pathogenic *Listeria* spp. that are isolated at a high rate requires further research to explain. Here, we performed the comparative genome sequence analysis of *L. innocua* recovered from dairy and dairy products in Egypt.

As indicated from WGS, we observed absence of antimicrobial resistance genes except FosX. This gene that confers resistance to fosfomycin has been identified in previous studies from *Listeria* spp. (Scortti et al., 2018; Wilson et al., 2018; Parra-Flores et al., 2022). In both pathogenic and non-pathogenic *Listeria* spp., a *Fos*X enzyme that is expressed by the *fos*X gene confers intrinsic fosfomycin resistance. However, when infected with pathogenic *L. monocytogenes*, the host signals activate *prf*A virulence regulons, *hpt*, and *prf*A and epistatically promote greater fosfomycin influx into the bacterial cell, suppressing *fos*X-mediated resistance (Scortti et al., 2018). Current whole genome sequenced *L. innocua* isolates showed absence of both *prf*A virulence regulons, *hpt* and *prf*A, indicating that fosfomycin treatment would not be effective under infection circumstances. ResFinder results also revealed absence of other antimicrobial resistance genes in isolates from this study. The few antimicrobial resistance genes found in these *L. innocua* isolates were generally consistent with past findings (Hof, 1991; Charpentier and Courvalin, 1999), and the most recent study conducted in the United States between 2010 and 2021 (Jorgensen et al., 2021; Hanes and Huang, 2022). As reported in these studies, most *L. monocytogenes* as well as strains of other *Listeria* spp., were found to be susceptible to a wide range of antimicrobials and there was no increase in antimicrobial resistance genes except for cephalosporin, fosfomycin, lincosamide and tetracycline resistance genes. Despite the few occurrences of antimicrobial resistance genes among the examined *L. innocua* isolates, which is considered a good sign for infection treatment, the spread of antimicrobial resistance among different bacterial species remains a major problem in Egyptian dairy farms (Tartor et al., 2021; Badawy et al., 2022). Further studies on larger sets would be helpful to determine the state of antimicrobial resistance in *Listeria* from milk and dairy products, since high antimicrobial resistances among different bacterial species from dairy farms in Egypt prevail.

*L. monocytogenes* pathogenesis requires the coordinated expression of six genes, namely *prf*A, *plc*A, *hly*, *mpl*, *act*A, and *plc*B, which are primarily assembled in the 9 kb *Listeria* Pathogenicity Island 1 (LIPI-1) (Vázquez-Boland et al., 2001). The *prf*A gene encodes a transcriptional activator, *prf*A, which directly or indirectly induces the transcription of over 140 genes, including the other five genes found in LIPI-1 (Paramithiotis et al., 2014). Both *plc*A and *plc*B encode phospholipases C (phosphatidylinositol and phosphatidylcholine), which, in conjunction with listeriolysin O (LLO), protect *Listeria* from cytoplasmic phagosomes (Schlüter et al., 1998). The *mpl* encodes a zinc-metalloprotease required for pro-*plc*B maturation (Raveneau et al., 1992), and *act*A is a multifunctional virulence factor (Travier et al., 2013). Our findings revealed the existence of 13 virulence genes among the examined isolates, belonging to adherence (*fbp*A, and *lap*), invasion (*iap*/*cwh*A, *gtc*A, and *lpe*A), surface protein anchoring (*lsp*A), peptidoglycan modification (*oatA*, and *pdgA*), intracellular survival (*lpl*A1, and *prs*A2) and heat shock proteins (*clp*C, *clp*E, and *clp*P). These genes that encode for minor or accessory virulence factors, were detected in most *L. innocua* strains. Nevertheless, *L. innocua* isolates carrying the above virulence profile were found associated with clinical cases in different hosts such as ruminants (Matto et al., 2022), and birds (Moura et al., 2019). Fortunately, none of the important LIPI-1 genes were found in the currently studied *L. innocua*. Virulence genes such as *hly*, which is responsible for the β-hemolysis associated with *L. monocytogenes* and some atypical hemolytic *L. innocua* (Johnson et al., 2004), were not identified in these *L. innocua* isolates.

*Listeria* spp. strains might acquire genes *via* mobile genetic elements, enhancing their capacity to endure and colonize a variety of food processing environments. These resistance and adaptation genes can increase tolerance of *Listeria* to a variety of stressors, such as sanitizers, antimicrobials, and environmental toxins, as well as extremes in salinity, acidity, and temperature. Transfer of such genes may also explain the widespread nature of *Listeria* in both natural and agricultural settings (Korsak and Szuplewska, 2016; Hingston et al., 2017; Pöntinen et al., 2017). Several genes that are responsible for the survivability and pathogenicity of *Listeria* spp., are plasmid mediated which can be transferred within and between *Listeria* spp. (Kuenne et al., 2010; Schmitz-Esser et al., 2021). Based on their replication protein *rep*A, *Listeria* plasmids mostly belong to groups 1 and 2 which are commonly known in plasmid database (PLSDB) as *rep*25 and *rep*26, respectively. The examined *L. innocua* isolates were found possessing plasmids of *rep*25 replicon type; this replicon type was previously identified in *L. monocytogenes* strain 2015TE24968, causing a severe listeriosis outbreak in Central Italy (Orsini et al., 2018) and environmental strain N1-011A (GenBank accession number CP006611.1) isolated in the United States. The ATP-dependent protease (*clp*L), which increases heat resistance, was initially discovered in *L. monocytogenes* harboring plasmid-borne *clp*L. It was revealed that *clp*L gene introduction into a naturally heat-sensitive strain of *L. monocytogenes* greatly boosted the recipient strain’s heat resistance, but plasmid-borne *clp*L deletion resulted in dramatically lower heat resistance in the wild-type strain (Pöntinen et al., 2017). To our knowledge, this is the first time that plasmid-borne *clp*L has been reported in *L. innocua*. The presence of such plasmid-borne *clp*L in *L. innocua* isolated from dairy products may allow them to survive at high processing temperatures, necessitating a stricter food product control protocol. It is also critical to understand the role of these plasmid-mediated genes in the pathogenicity and persistence of *Listeria* in challenging and *in vitro* environments. Fortunately, due to the similarities in ecology, genome, and physiological traits between both *L. monocytogenes* and *L. innocua*, researchers are able to control *L. monocytogenes* by using *L. innocua* surrogates and identifying hidden metabolomic tools for surviving stressors (He et al., 2021; Wu et al., 2021). Considering this, further research into the metabolic changes caused by the plasmid-borne *clp*L gene in *L. innocua* will be required.

WGS has become a powerful tool used for routine epidemiological surveillance of infectious diseases, outbreak investigations, and tracing transmission routes (Oakeson et al., 2018; Brown et al., 2021). MLST and SNP-based phylogeny are frequently used for bacterial subtyping, that enables precise source attribution and identification of the origin of pathogen associated-outbreaks (Stessl et al., 2021). In the present study, the 5 *L. innocua* isolates sourced from milk and yoghurt were assigned to the same sequence type, ST-1085. SNP-based phylogeny confirmed the MLST findings of the sequenced isolates, and few SNP differences (less than 10 SNPs) were identified among isolates indicating the circulation of the same *L. innocua* clone in raw milk and dairy product. Comparing our isolates to the global lineages of *L. innocua*, our isolates showed close similarity with *L. innocua* isolates (422-1,091 SNPs) that were recovered from food and the environment. This highlights the importance of WGS-based phylogeny for monitoring the potential sources of foodborne pathogens.

In conclusion, the sequencing results revealed that current isolates of *L. innocua* lacked the *Listeria* Pathogenicity Island 1 (LIPI-1) gene, the *hly* gene, and any antimicrobial resistance genes, except for the *fos*X gene. However, the absence of *fos*X gene suppressing regulons, *hpt*, and *prf*A suggests that fosfomycin treatment would be ineffective under infection conditions. Furthermore, the 5 *L. innocua* isolates possessed 13 virulence genes involved in adhesion, invasion, surface protein anchoring, peptidoglycan degradation, intracellular survival, and heat stress. Though these genes encode for minor or accessory virulence factors, the close genetic relationship with *L. monocytogenes* and the potential for horizontal virulence gene transfer raises the possibility of evolution of virulent *L. innocua* strains. The ATP-dependent protease (*clp*L), mediating elevated heat resistance of strains which carry it, was reported for the first time in the currently studied *L. innocua*, specifically that *clp*L was carried by plasmids. The first documentation of *clp*L-carrying plasmids in *L. innocua*, along with the common virulence and antimicrobial resistance genes, and the ongoing various genetic mechanisms of virulence transfer among *Listeria* and other genus, could endanger current industrial processing and preservation protocols and present health risks from dairy products. Therefore, ongoing genomic studies are necessary to identify these alarming genetic changes and research is needed to develop and test preventive and control measures.

## Data availability statement

The datasets presented in this study can be found in online repositories. The names of the repository/repositories and accession number(s) can be found at: https://www.ncbi.nlm.nih.gov/, Bioproject ID. PRJNA921817.

## Author contributions

HR, MA-A, and CJ conceived and designed the study. HR performed the microbiology work and whole-genome sequencing of isolates, analyzed the genome data, and wrote the original draft of the manuscript. AS and ME participated to the analysis of WGS data. IS contributed to the data analysis and manuscript drafting. MY and LH participated to microbiology work. AA contributed to the data analysis. HR, MA-A, MB, JF, and CJ secured funding and provided project administration. HR, MA-A, AS, ME, IS, AA, MB, JF, and CJ reviewed and edited the manuscript. All authors contributed to the article and approved the submitted version.

## Funding

This work has been funded by the U.S. Department of Agriculture (USDA) project 6040-32000-079-00-D.

## Conflict of interest

The authors declare that the research was conducted in the absence of any commercial or financial relationships that could be construed as a potential conflict of interest.

## Publisher’s note

All claims expressed in this article are solely those of the authors and do not necessarily represent those of their affiliated organizations, or those of the publisher, the editors and the reviewers. Any product that may be evaluated in this article, or claim that may be made by its manufacturer, is not guaranteed or endorsed by the publisher.

## Footnotes

^1^ https://www.bioinformatics.babraham.ac.uk/projects/fastqc/

^2^ https://usegalaxy.org/

^3^ https://github.com/tseemann/abricate

^4^ http://sourceforge.net/projects/brig

^5^ http://mjsull.github.io/Easyfig/

^6^ https://www-is.biotoul.fr/blast.php

^7^ http://bigsdb.web.pasteur.fr/listeria/

^8^ https://github.com/tseemann/snippy
